# Acetate modulates the inhibitory effect of *Lactobacillus gasseri* against the pathogenic yeasts *Candida albicans* and *Candida glabrata*

**DOI:** 10.15698/mic2023.04.795

**Published:** 2023-03-21

**Authors:** Nuno A. Pedro, Gabriela Fontebasso, Sandra N. Pinto, Marta Alves, Nuno P. Mira

**Affiliations:** 1 iBB, Institute for Bioengineering and Biosciences, Instituto Superior Técnico – Department of Bioengineering, Universidade de Lisboa, 1049-001 Lisboa, Portugal.; 2 Associate Laboratory i4HB—Institute for Health and Bioeconomy at Instituto Superior Técnico, Universidade de Lisboa, Av. Rovisco Pais, 1049-001 Lisboa, Portugal.; 3 CQE-Centro Química Estrutural, Instituto Superior Técnico, Universidade de Lisboa, Av. Rovisco Pais, 1049-001 Lisboa, Portugal.

**Keywords:** lactobacilii-Candida interference, vaginal lactobacilii, vaginal candidiasis, probiotics, lactobacillus gasseri, candida physiology

## Abstract

The exploration of the interference prompted by commensal bacteria over fungal pathogens is an interesting alternative to develop new therapies. In this work we scrutinized how the presence of the poorly studied vaginal species *Lactobacillus gasseri* affects relevant pathophysiological traits of *Candida albicans* and *Candida glabrata*. *L. gasseri* was found to form mixed biofilms with *C. albicans* and *C. glabrata* resulting in pronounced death of the yeast cells, while bacterial viability was not affected. Reduced viability of the two yeasts was also observed upon co-cultivation with *L. gasseri* under planktonic conditions. Either in planktonic cultures or in biofilms, the anti-*Candida* effect of *L. gasseri* was augmented by acetate in a concentration-dependent manner. During planktonic co-cultivation the two *Candida* species counteracted the acidification prompted by *L. gasseri* thus impacting the balance between dissociated and undissociated organic acids. This feature couldn't be phenocopied in single-cultures of *L. gasseri* resulting in a broth enriched in acetic acid, while in the co-culture the non-toxic acetate prevailed. Altogether the results herein described advance the design of new anti-*Candida* therapies based on probiotics, in particular, those based on vaginal lactobacilli species, helping to reduce the significant burden that infections caused by *Candida* have today in human health.

## INTRODUCTION

Candidiasis are infections caused by pathogenic yeasts of the *Candida* genus and account around 50 to 70% of the reported fungal infections worldwide [1]. Infections caused by *Candida* are more frequent in the oral or in the vaginal mucosa, but in more serious cases, often life-threatening, these yeasts disseminate in the bloodstream and colonize major organs [2, 3]. Due to their high mortality rates, aggressiveness and recurrence, infections caused by *Candida* have a very high societal impact [4]. The occurrence of systemic candidiasis is most frequently observed upon immunosuppression (e.g., in patients undergoing chemotherapy or in the elderly), but vaginal candidiasis is common among the healthy female population. Women are described to suffer two to three episodes of vaginal candidiasis during their life-time, with a significant proportion (5 to 10%) suffering from recurrent infections, a condition known as recurrent vulvovaginal candidiasis [5, 6]. The more relevant species causative of candidiasis, both superficial and systemic, is *Candida albicans* but the incidence of infections caused by non-albicans *Candida* species (also called as NACS) is increasing, in some geographies prominently [7, 8]. Infections caused by NACS are worrisome as these species are usually very resilient to commonly used antifungals and the underlying infections have poorer outcomes for patients, compared to infections caused by *C. albicans* [9, 10]. Among NACS, *Candida glabrata* is usually the most prevalent species, in part due to its innately high tolerance to azoles and extreme genomic plasticity that, among other traits, prompts fast adaptive responses to the challenging environment of infection sites [9, 11].

A distinguishing aspect of the pathophysiology of *Candida*, compared with other human-infecting fungi, is that these species are part of the microbiota of various niches, even in the absence of disease [12-14]. Indeed, *C. albicans* has been identified as a true gut symbiont based on its consistent identification in resident microbial populations of this niche [12, 13]. *C. glabrata* has also been identified in the gut microbiome, however, not consistently, thus remaining to be elucidated whether it is a true symbiont or a transient passenger [15, 16]. *C. albicans* and *C. glabrata* have also been identified in the vaginal mycobiome of “healthy” women, using both culture-dependent and independent methods [17-19]. However, these yeasts are not ubiquitously observed in all samples suggesting that the presumed “healthy” women could be asymptomatic carriers and leaving open whether or not the vaginal tract is a primary site of colonization of *Candida*. While in the past not much attention was given to these commensal populations of *Candida*, in the recent years it has been demonstrated that they can be reservoirs for dissemination, especially in the gastrointestinal (GI) tract [3, 12].

Besides *Candida* a plethora of other species compose the vaginal microbiome, their identity and abundance differing with the anatomic site (for example, prominent differences were observed between the cervix or the uterus), with age, habits or race [20-22]. Despite the inter-individual variation, it is clear that the vaginal microbiome is dominated by lactobacilli with *Lactobacillus iners, Lactobacillus crispatus, Lactobacillus jensenii* and *Lactobacillus gasseri* being most abundant [23, 24]. Perturbation of the vaginal microbiome was linked to adverse gynaecologic/obstetric outcomes including preterm birth [25], mucosal inflammation [26] or infections caused by HPV [27], HIV [28] or bacteria [22, 29]. Studies examining the vaginal microbiome of asymptomatic women or of patients with diagnosed vaginal candidiasis obtained conflicting results: some reported decreased abundance of the lactobacilli population; others report only changes in the species profile (for example, unusual predominance of *L. iners*); and others don't report any alterations to the habitual lactobacilli-enriched flora [17, 19, 30-37]. Although it is not totally clear whether vaginal lactobacilli provide protection against candidiasis *in vivo*, the potential of those species in inhibiting growth and relevant pathogenic traits of *Candida in vitro* has been well reported [38-40]. These observations opened the door to the use of probiotics based on lactobacilli as possible anti-*Candida* treatments with results pointing to a positive contribution in the prevention of relapse and avoidance of recolonization [41-43]. However, most of these probiotic cocktails were developed using lactobacilli species not indigenous to the vaginal tract, likely due to the poor knowledge available concerning their genetics and physiology [41, 44]. Intestinal lactobacilli species (which differ from those found in the vaginal tract) have also been found to restrain growth and virulence of *Candida in vitro* and in infection models [45-47].

Resulting from this interest in the exploitation of the lactobacilli microflora as potential anti-*Candida* agents some studies examined this interference in more detail (as reviewed in [47-49]). Usually, the role of lactobacilli as drivers of vaginal health was attributed to lactic acid production which maintains an acidic vaginal pH and restrains bacterial growth [50-52]. However, the acidophilic nature of yeasts, including *Candida* spp., along with the demonstration that at pH of 4 (the usual pH of a vaginal fluid dominated by lactobacilli [53]) physiologically relevant concentrations of lactic acid don't inhibit growth of *C. albicans* or *C. glabrata* [54], suggests that lactic acid may play a minor role in restraining *Candida*. Interestingly, it has been shown that lactate promotes the evasion of *Candida* cells from the immune system by inducing β-glucan masking and reducing macrophage recruitment [55], suggesting that these yeasts evolved adaptive responses to allow them to cope with the presence of this acid anion in the environment in a manner that favors colonization and, eventually, infection. Additionally, lactic acid was found to be a potent immunomodulator, reducing production of inflammatory cytokines by epithelial cells and thereby restraining the inflammatory response [56-58]. More recently, the anti-*Candida* potential of some lactobacilli species was attributed to the production of 1-acetyl-β-carboline [59], however, the only vaginal lactobacilli species studied was *L. gasseri* and the authors concluded that under their experimental setting supernatants obtained from culturing this bacterium inhibited filamentation of *C. albicans*, but not growth [59]. Other studies also demonstrated reduced filamentation and growth of *C. albicans* when cultivated in medium supplemented with supernatants obtained from *L. gasseri, L. jensenii* or *L. crispatus* cultures, although strain-to-strain variation was observed, especially for *L. gasseri* strains [40]. Other described anti-*Candida* effects attributed to lactobacilli involve the reduction in the ability of the yeasts to bind to epithelial cells due to a higher bacterial affinity for epithelial receptors, secretion of biosurfactants [60-63] or the production of bacteriocins [64, 65]. However, no specific molecular players mediating these responses have been identified.

In this work we examined growth, physiology and virulence traits of *C. albicans* and *C. glabrata* when co-cultivated with the poorly studied vaginal species *L. gasseri* under planktonic or biofilm-forming conditions. This combined approach is a distinct aspect of our work since the majority of studies addressing this interaction used the cultivation of the yeasts in the presence of supernatants produced by vaginal bacterial cultures and not directly the cell-cell interaction. Besides assessing relevant physiological aspects of the interaction established between these two species, we have also uncovered the role of acetate as a positive modulator of the interference of *L. gasseri* over *Candida* even in concentrations similar to those found in a vaginal microflora dominated by lactobacilli.

## RESULTS

### Co-cultivation of *C. glabrata* and *C. albicans* with *L. gasseri* results in decreased viability of the yeasts, either under planktonic or biofilm-forming conditions

**Figure 1:**
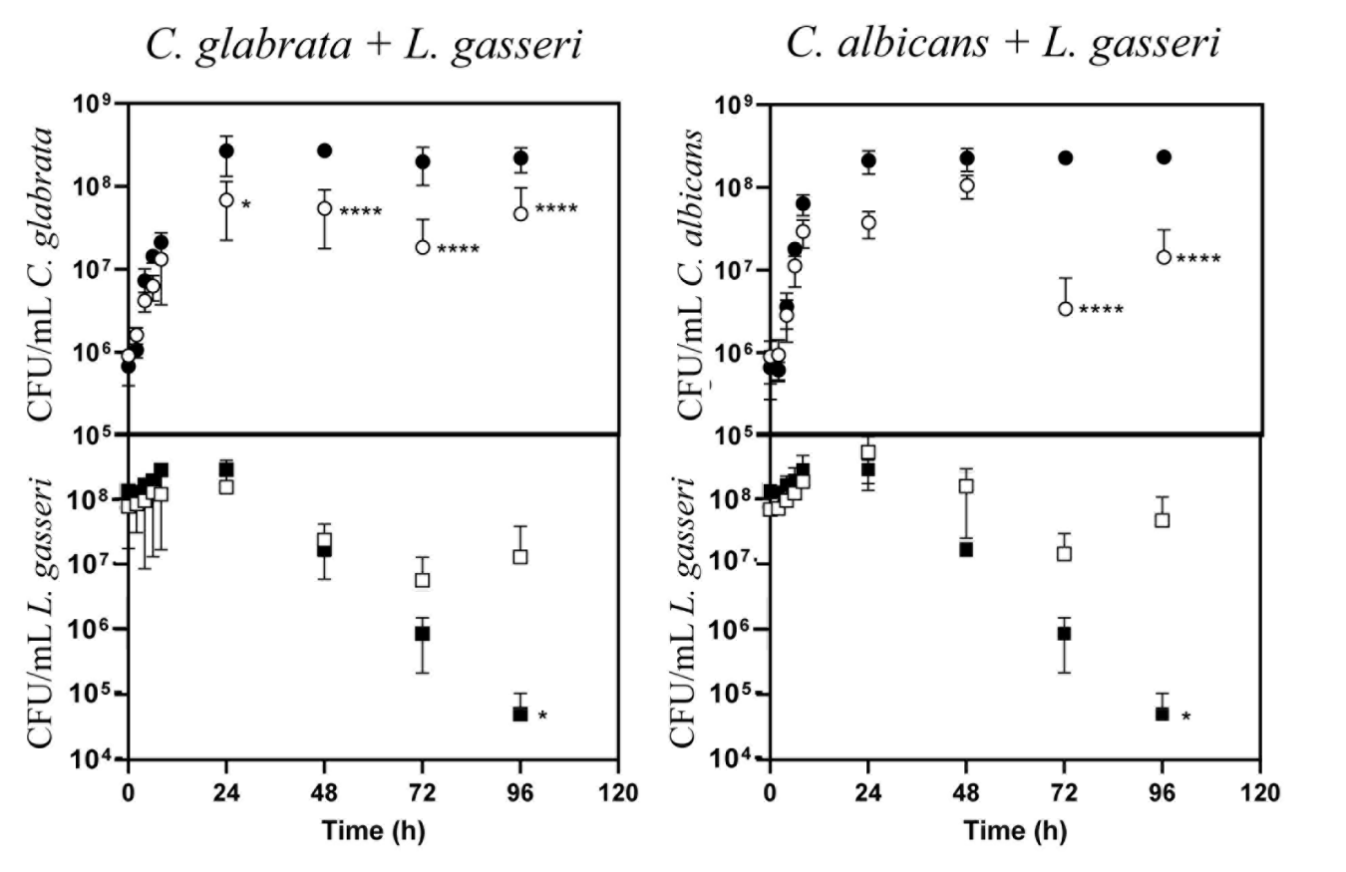
FIGURE 1: Cellular viability of *C. glabrata, C. albicans* and *L. gasseri* along single or co-cultivation in MRS medium. After inoculation, cells of *C. albicans* (○,•), *C. glabrata* (○,•) or *L. gasseri* (□,▪) were cultivated at 37°C and 100 rpm for 96 h with growth of the different species being accompanied based on cellular viability, as detailed in materials and methods. Filled symbols correspond to the samples taken during single-species cultivation while open symbols correspond to the samples taken during co-cultivation.

The majority of the studies that examined the interference established between the vaginal species *L. gasseri* and *Candida* focused on the inhibitory effect prompted by supernatants obtained from bacterial cultures [61, 66, 67], usually obtained in MRS, the canonical growth medium used for lactobacilli [68, 69]. Considering that *in vivo* these two species are in close contact, we focused on their direct co-cultivation under planktonic and biofilm-forming conditions. To examine planktonic growth, the two *Candida* species (*C. albicans* and *C. glabrata*) and *L. gasseri* ATCC33323 were cultivated in liquid MRS for 96 h (**Fig. 1**). To simulate the higher abundance of lactobacilli in the vaginal microflora compared with the one of *Candida* [70-73], these co-cultivations were started using ∼100x more bacterial than yeast cells (∼10^8^ CFUs/mL *L. gasseri* compared to 10^6^ CFUs/mL of *C. albicans* or *C. glabrata*; **Fig. 1**). Under the experimental conditions used for the co-cultivation (100 rpm of agitation and 37°C) the yeast cells resumed growth immediately after re-inoculation and maintained it until 24 h after which they entered stationary phase (**Fig. 1**). The bacterial population also increased, although much less, likely due to a higher number of inoculated cells, compared with the one use for *Candida* (**Fig. 1**). Consistent with co-cultivation being a more competitive and challenging environment, the growth rate of *C. glabrata* and *C. albicans* in the presence of *L. gasseri* decreased 32% and 33% respectively, compared to the values observed in single culture (0.25 h^-1^ obtained in single culture of *C. glabrata*, compared to 0.17 h^-1^ in co-culture; 0.24 h^-1^ obtained in single culture of *C. albicans*, compared to 0.16 h^-1^ obtained in co-culture). Besides a reduction in the growth rate, it was also noticeable that co-cultivation with *L. gasseri* induced a prominent decrease (ranging between 53 and 98%) in the cellular viability of *C. albicans* and *C. glabrata*, this being considerably more prominent for the first species (**Fig. 1**). No significant reduction in viability was observed in the two single cultures of *Candida* indicating that the loss of viability observed in the co-culture setting is a direct effect of the presence of *L. gasseri* (**Fig. 1**). Differently, co-cultivation increased cellular viability of *L. gasseri* up to 10^7^ CFUs/mL, compared to 10^5^ CFUs/mL attained in single-culture (**Fig. 1**). In agreement with our results, reduced viability of *L. gasseri* cells upon short-medium term cultivation in MRS have been reported in other studies, presumably due to autolysis [74-79]. In order to exclude strain-dependent effects, we have repeated the co-cultivations using four vaginal *C. glabrata* and *C. albicans* strains and in both cases it was clear that co-cultivation with *L. gasseri* resulted in reduced viability of the yeast strains (see results in Supplementary Fig. S1). However, it was interesting to observe that the decrease in viability of the vaginal *Candida* strains imposed by co-cultivation with *L. gasseri* was smaller than the one obtained with the reference strains (compare results in Supplementary Fig. S1 and **Fig. 1**).

**Figure 2:**
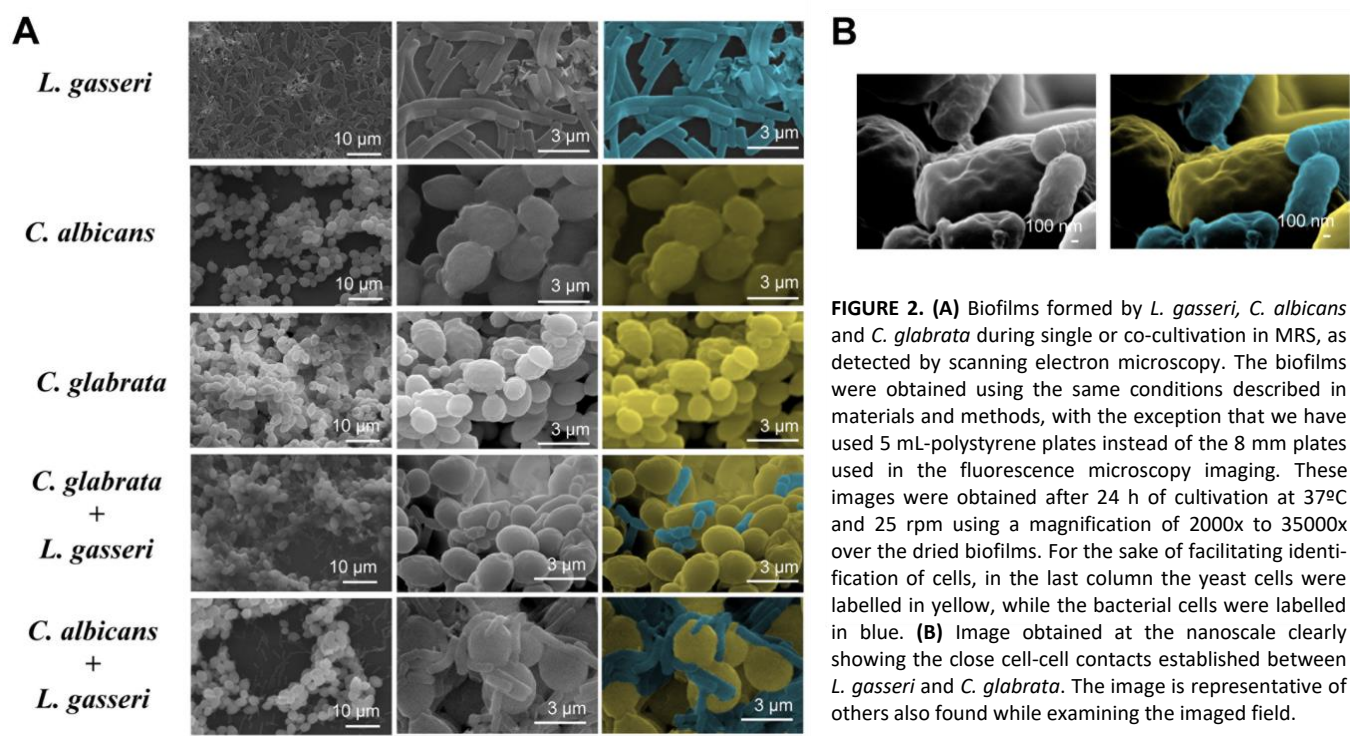
FIGURE 2. **(A)** Biofilms formed by *L. gasseri, C. albicans* and *C. glabrata* during single or co-cultivation in MRS, as detected by scanning electron microscopy. The biofilms were obtained using the same conditions described in materials and methods, with the exception that we have used 5 mL-polystyrene plates instead of the 8 mm plates used in the fluorescence microscopy imaging. These images were obtained after 24 h of cultivation at 37°C and 25 rpm using a magnification of 2000x to 35000x over the dried biofilms. For the sake of facilitating identification of cells, in the last column the yeast cells were labelled in yellow, while the bacterial cells were labelled in blue. **(B)** Image obtained at the nanoscale clearly showing the close cell-cell contacts established between *L. gasseri* and *C. glabrata*. The image is representative of others also found while examining the imaged field.

**Figure 3:**
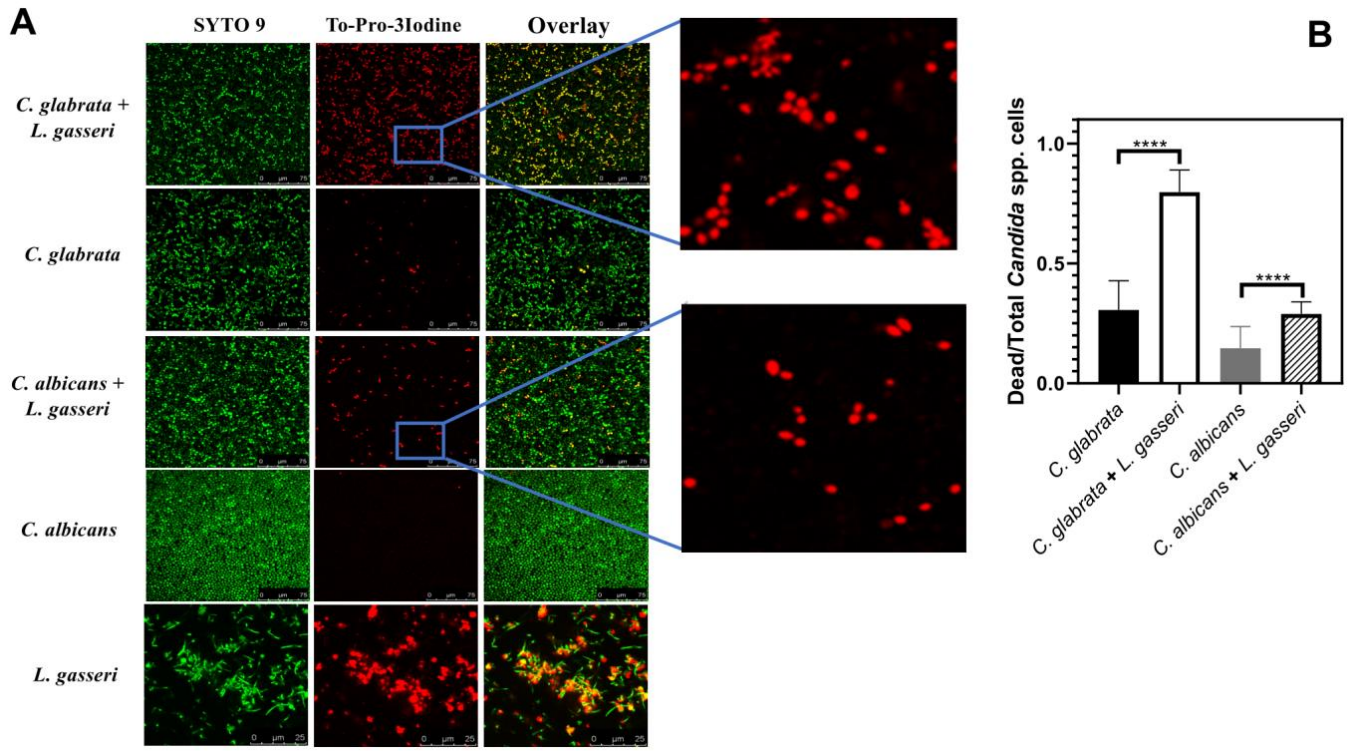
FIGURE 3. **(A)** Live/dead imaging of cells in single-species or in mixed biofilms formed by *L. gasseri, C. albicans* or *C. glabrata* after 24 h of cultivation, at 37°C and 25 rpm, in MRS. The insert details the labelling of the dead yeast cells in the mixed biofilms. The images presented are representative of a set taken from the biofilms in three replica experiments performed; *Candida* spp. single and mixed biofilms scale bar corresponds to 75 μm while *L. gasseri* single biofilm scale bar corresponds to 25 μm; **(B)** Quantification of the number of dead *Candida* cells in the single-species or in the multi-species biofilms formed, based on quantification of the number of red-labelled yeast cells in all pictures taken from the biofilms, compared to the total number of *Candida* cells in the field (corresponding to green-labelled cells). For this quantitative analysis more than 1000 yeast cells were imaged in each condition. Statistical significance was calculated using one-way ANOVA (****p-value below 0.0001).

To test the effect of co-cultivation under sessile conditions the same experimental setting was used with the difference that *C. albicans, C. glabrata* and *L. gasseri* were cultivated in 8-well microplates. After 24 h of cultivation it was possible to observe that *C. albicans* and *C. glabrata* formed a mixed biofilm with *L. gasseri* involving very close cell-to-cell contacts, as shown by the microscopy SEM images depicted in **Fig. 2**. Single-species biofilms formed by *C. albicans* exhibited what appeared to be a multi-layered structure, while those formed by *C. glabrata* appeared more disperse (**Fig. 3**). These differences in structural organization of the biofilms formed by these two yeasts is consistent with results reported in other studies [80-83]. No significant differences were obtained concerning height of the single-species and multi-species biofilms, with the exception of the biofilms formed by *L. gasseri* alone that were considerably thinner (Supplementary Fig. S2). To understand what could be the outcome of the formation of these mixed biofilms in terms of viability, we took advantage of SYTO9 and TO-PRO3 iodide labelling that allowed us to differentiate, the yeast from the bacterial cells, while also distinguishing viable from non-viable cells directly in the biofilm [84]. In **Fig. 3** we show the results of this labelling in single- and in multi-species biofilms. The results clearly demonstrate that the proportion of non-viable cells (labelled in red) was much higher in the mixed-biofilms than in the single-species ones, this effect being clearly more evident in the mixed biofilms formed between *L. gasseri* and *C. glabrata* (**Fig. 3**). Closer inspection of the images shows that these non-viable red-labelled cells correspond almost entirely to the yeasts while no significant loss of viability was observed for the *L. gasseri* cells (**Fig. 3**). To get a more quantitative view, we imaged in close detail a set of pictures (corresponding to more than 1000 yeast cells per condition) and found that the number of non-viable *C. glabrata* cells was about 2.6-fold higher in the mixed-biofilms than in the biofilms formed in the absence of the bacterium (**Fig. 3**). In the case of *C. albicans* the number of non-viable cells in the mixed-biofilm increased roughly 2-fold (**Fig. 3**). A striking labelling of TO-PRO-3 iodide was observed in the single-species *L. gasseri* biofilms visualizing at higher magnification that the labelling corresponds to what appears to be the extracellular matrix (**Fig. 3** and a magnification shown in Supplementary Fig. S3). Upon entry into microbial cells, TO-PRO-3 iodide is described to bind nucleic acids [84, 85] and thus it is possible that the observed labelling results from accumulated extracellular DNA (eDNA), as this was described to occur in biofilms formed by other lactobacilli species [86-88]. Notably, such labelling pattern was not detected in the single-species biofilms formed by the two *Candida* species, albeit eDNA has been reported in the extracellular matrix of *C. albicans* biofilms [89].

### While in co-cultivation with *L. gasseri, C. albicans* and *C. glabrata* buffer the acidification prompted by the bacterium with consequences in the equilibrium of ionizable species like lactic and acetic acids

**Figure 4:**
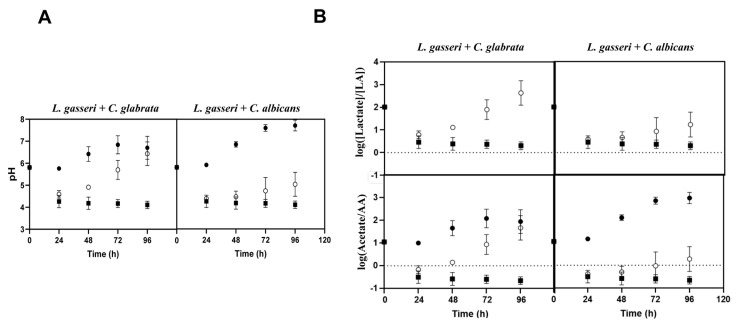
FIGURE 4. **(A)** Variation of medium pH during cultivation of *L. gasseri* (□, ▪) in MRS alone or in combination with C. *glabrata* or *C. albicans* (○, •), under the same conditions as those used to obtain the growth curves shown in Fig. 1. In panel **(B)** the variations in the ratio of lactate/ lactic acid (LA)/ and acetate/acetic acid (AA) is shown along the single-species or co-cultivation settings, as estimated by the Handerson-Hasselbach equation and using the pHs determined in panel A, a pKa of 3.8 for lactic acid and a pKa of 4.76 for acetic acid. Filled symbols correspond to the samples taken during single-species cultivation while open symbols correspond to the samples taken during co-cultivation. The results presented are representative of, at least, three independent replicas.

Taking into account that co-cultivation increased viability of *L. gasseri*, while it decreased viability of the two *Candida* species (**Fig. 1**), and that the high autolysis of *L. gasseri* in MRS was associated to the low pH of the medium (presumably due to the lactic acid formed) [77], we decided to monitor the pH achieved in single and in co-cultures. HPLC analyses of the broth confirmed the expected production of lactic acid by *L. gasseri* in amounts that ranged between 10 g/L in the single-culture to approximately 5 g/L in the co-culture (Supplementary Fig. S4). The lower amount of lactic acid produced in the co-cultures was consistent with a more rapid depletion of glucose in the fermentation medium, likely resulting from the yeast's metabolic activity (Supplementary Fig. S4). As expected, no production of lactic acid was observed in the supernatants obtained from single cultures of the two *Candida* species, only an accumulation of ethanol was detectable (results not shown), which is compatible with the microaerophilic setting used. Concomitant with the production of lactic acid, a continuous acidification of the fermentation broth was observed, both in the single and in the co-cultivation settings (**Fig. 4A**). While in the single-cultures of *L. gasseri* the acidification persisted along the entire 96 h time frame and reaching a final pH of about 4, in the co-culture the pH started to increase after an initial drop (**Fig. 4A**). These observations suggest that the metabolic activity of the two *Candida* species buffers the acidification prompted by the accumulation of lactic acid, this buffering capacity being higher for *C. glabrata* than for *C. albicans* (final pH of the co-cultures was of 6.8 and 5, respectively; **Fig. 4A**). The same capacity of *C. glabrata* and *C. albicans* to alkalinize the broth when in co-cultivation with *L. gasseri* was observed for the tested vaginal strains (results not shown).

One of the factors imparted by the differences in pH of the broth registered during the single or co-cultivation of *C. albicans*/*C. glabrata* with *L. gasseri* is the distribution between the dissociated (RCOO^-^) and undissociated form (RCOOH) of lactic acid, as well as of other ionizable species accumulated in the broth, necessarily depending on their corresponding p*Ka* values. Using the Handerson-Hasselbach equation to estimate the ratios between the amounts of lactate and lactic acid achieved during single or co-cultivation with *Candida*, it is clear that most of the lactic acid accumulated in the broth in the co-culture was dissociated (**Fig. 4B**). In the single-cultures of *L. gasseri* lactate also prevailed, however, the amount of undissociated lactic acid was considerably higher than the one present in the broth of co-cultures (**Fig. 4B**). Besides lactic acid, we also computed the ratio of dissociated and undissociated acetic acid since the MRS medium contains ∼60 mM of sodium acetate supplied as a sodium source (**Fig. 4B**). In this case, there was a marked difference between the result obtained in single-cultures of *L. gasseri* and in co-cultures, as undissociated acetic acid clearly predominated in the single-culture, while in the co-culture acetate prevailed (**Fig. 4B**). These observations show that the co-cultivation of *C. glabrata* or *C. albicans* with *L. gasseri* modulates important aspects of the composition of the supernatant including its pH and, consequently, the acid-base equilibrium of ionizable species like lactic and acetic acids.

### Acetate augments anti-*Candida* activity prompted by *L. gasseri* cells

**Figure 5:**
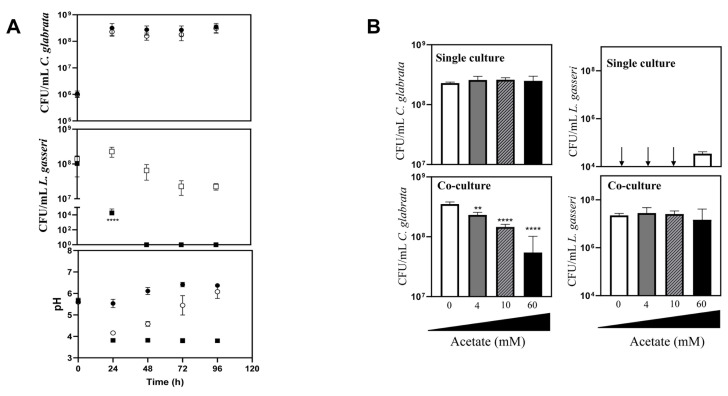
FIGURE 5: The acetate present in MRS medium potentiates the inhibitory effect of *L. gasseri* over *C. glabrata* cells. **(A)** Cellular viability and medium pH during single or co-cultivation of *C. glabrata* (○, •) with *L. gasseri* (□, ▪) in MRS medium having 60 mM sodium chloride as the sodium source (instead of the normally used sodium acetate). The cells were cultivated alone or in the presence of each other, under the same conditions described in Fig. 1. Filled symbols correspond to the samples taken during single-species cultivation while open symbols correspond to the samples taken during co-cultivation. **(B)** Cellular viability of *C. glabrata* and *L. gasseri* after 96 h of cultivation in single culture or in co-culture in MRS media having increasing concentrations of acetate. Note the decreasing viability of the yeast cells as the concentration of acetate increases. The results shown in this panel concerning cellular viability of the different microbial species were taken from the full growth curves that are shown in Supplementary Fig. S3. Statistical significance of the differences found in the presence or absence of acetate were calculated using one-way ANOVA (*p-value below 0.05; **p-value below 0.01; ***p-value below 0.001; ****p-value below 0.0001).

Our results shown in **Fig. 4B** demonstrate that in co-cultures of *L. gasseri* with *C. albicans* or *C. glabrata* the sodium acetate supplied in the MRS medium prevails in the acetate form. Considering that acetate has been demonstrated to induce the expression of bacteriocins in several Gram positive species [90, 91], we hypothesized whether it could also modulate the anti-*Candida* effect exhibited by *L. gasseri*. For this we co-cultivated *L. gasseri* with *C. glabrata* in MRS medium under the same conditions used before but replacing sodium acetate by the same amount of sodium chloride. This replacement resulted in an accelerated loss of viability of *L. gasseri* cells when in single-culture, a phenotype that was rescued when the bacterial cells were cultivated in the presence of the yeasts (**Fig. 5A**). Remarkably, despite the maintenance in the viability of *L. gasseri* population, no significant loss of viability of *C. glabrata* could be detected in the co-cultures performed in MRS with NaCl (**Fig. 5A**). Notably, the capability of acetate to enhance the ability of *L. gasseri* cells to reduce viability of *C. glabrata* cells was concentration dependent and still detectable at concentrations as low as 4 mM (**Fig. 5B**, Supplementary Fig. S5). Consistent with the results observed for *C. glabrata*, co-cultivation of *L. gasseri* with *C. albicans* cells in MRS medium having NaCl (and not sodium acetate) as the sodium source also resulted in incapability of the bacterium to induce loss of viability in the yeast cell population (Supplementary Fig. S6). The replacement of sodium acetate by increasing amounts of sodium chloride led to a reduction in production of lactic acid (up to a maximum of 50%) prompted by the bacterium (Supplementary Fig. S4B and C). While in single-cultures, this decrease can be attributed to the lower viability of the bacterial cells when growing in MRS-NaCl, compared to MRS (**Fig. 5A, Fig. 1**), in the co-cultures performed in MRS-NaCl or in MRS the viability of *L. gasseri* cells was identical and therefore the lower production of lactic acid is likely to result from the lower availability of carbon (**Fig. 5A, Fig. 1**). The capability of acetate to increase the anti-*Candida* potential of *L. gasseri* was also detected when a vaginal clinical strain of the bacterium was used (*L. gasseri* ISTLg47) confirming that the effect is not exclusive for the used reference strain (Supplementary Fig. S7).

### The effect of acetate in augmenting anti-*Candida* activity in *L. gasseri* cells is also observed under biofilm-forming conditions

**Figure 6:**
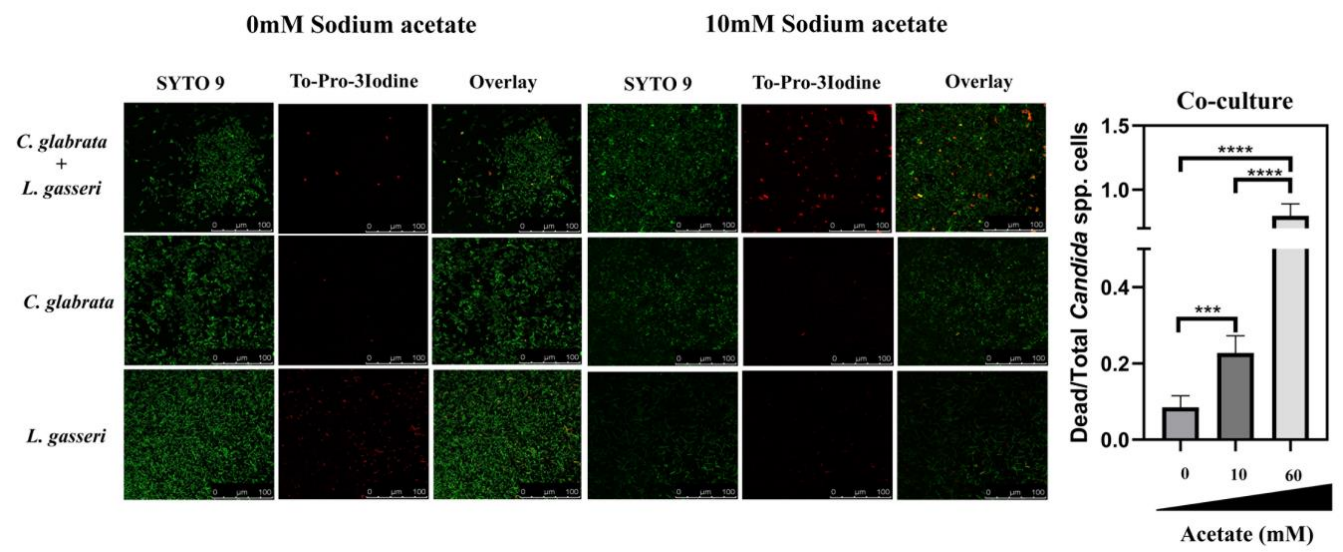
FIGURE 6. Effect of acetate on the capability of *L. gasseri* to induce loss of *C. glabrata* cellular viability in mixed biofilms. Biofilms formed by *L. gasseri* and *C. glabrata* after 24 h of single or co-cultivation in MRS medium with increasing amounts of acetate at 37°C and 25 rpm were imaged by fluorescence confocal microscopy to distinguish between live and dead cells, as detailed in materials and methods. The pictures presented are representative of a cohort obtained in three independent replicas and that were used to quantify the number of dead *C. glabrata* cells (labelled in red) compared to the total number of *C. glabrata* cells in the field (labelled in green and distinguishable from the bacterial cells based on their yeast-like morphology). All scale bars corresponds to 100 μm. The result of the quantification is shown in the chart presented at the right. The data presented concerning the number of *C. glabrata* cells in mixed biofilms formed in MRS having 60 mM acetate is the one shown in Fig.3. Statistical significance of the differences obtained in the number of dead yeast cells in the different conditions was calculated using one-way ANOVA (*p-value below 0.05; **p-value below 0.01; ***p-value below 0.001; ****p-value below 0.0001).

The above reported effect of acetate in enhancing the capability of *L. gasseri* cells to induce loss of viability in *C. albicans* and in *C. glabrata* prompted us to examine whether the same effect could also be detected in biofilms. For that, we have used the same experimental setting as used above to detect the formation of mixed biofilms between *L. gasseri* and *C. albicans* or *C. glabrata*, including the live-dead confocal microscopy imaging. The modulation of acetate concentration in the MRS medium did not significantly alter the height of the biofilms formed (Supplementary Fig. S2). On the other hand, a marked increase in the viability of *C. glabrata* and *C. albicans* in mixed biofilms formed with *L. gasseri* during cultivation in MRS without acetate was observed, markedly contrasting with the high loss of viability that was observed in the normal composition of this medium including 60 mM acetate (**Fig. 6** and Supplementary Fig. S8). Also as observed under planktonic conditions, this effect of acetate in augmenting the anti-*Candida* activity of *L. gasseri* cells was concentration-dependent (**Fig. 6** and Supplementary Fig. S5). Strikingly, the unusual TO-PRO-3-labelling registered in the biofilms formed by *L. gasseri* cells were no longer detected when acetate was removed from the medium (results not shown).

## DISCUSSION

In this work we focused on the interaction established between the poorly studied vaginal species *L. gasseri* and the pathogenic yeasts *C. albicans* and *C. glabrata*, both being frequent colonizers of the female vaginal tract. Other studies described the inhibitory potential of lactobacilli species, including *L. gasseri*, against *Candida* and other vaginal pathogens [39, 67, 92]. However, these studies don't explore co-cultivation describing instead relevant alterations in pathophysiological traits of *Candida* when cultivated in media supplemented with various amounts of supernatants obtained from bacterial cultures [61, 66, 67, 93]. The results shown herein demonstrate that the direct co-cultivation of *L. gasseri* with *C. albicans* and *C. glabrata* has outcomes that cannot be fully recapitulated by the bacterial supernatants themselves. For example, co-cultivation of *C. albicans*/*C. glabrata* with *L. gasseri* drastically altered the pH of the broth with consequences for the acid-base equilibrium of ionizable species like the organic acids acetic and lactic acid. In particular, we found that during co-cultivation, the two yeasts counteracted the prominent acidification of the medium promoted by the accumulation of lactic acid produced by *L. gasseri* cells. Consequently, the final pH of the co-cultures was ∼6 after 96 h, while in the single cultures of *L. gasseri* this pH was about ∼4.1. Prior studies have shown the capability of *C. albicans* to alkalinize the medium (via ammonium excretion) when using amino acids [94, 95] or carboxylic acids as carbon sources [94, 95]. *C. glabrata* has also been shown to alkalinize the medium while using amino acids as carbon sources but the identity of the buffering compound was not disclosed [96]. Notably, in these studies the alkalinization of the medium prompted by *C. albicans* or *C. glabrata* occurred under glucose limiting conditions, which is in line with the results we had obtained since the pH started to increase after 48 h, when no glucose was available in the broth (Supplementary Fig. S4). This capability of *C. albicans* and *C. glabrata* to induce alkalinization under acidic conditions has been linked with their increased ability to thrive in the highly acidic phagolysosome, favoring colonization and immune evasion [96, 97]. In *C. albicans* the alkalinization has also been shown to auto-induce hyphal morphogenesis [95]. It is possible that *in vivo*, when present in the vaginal tract, *Candida* cells can also counteract the acidification prompted by lactobacilli. Among other outcomes (such as the promotion of yeast-hyphae transition) this buffering also avoids the accumulation of undissociated organic acids in the environment that have a potent antimicrobial effect, also against *Candida* [54, 98, 99]. Besides lactic acid, acetic acid, 4-hydroxyphenylacetic, succinic, butyric and formic acids are other organic acids present in the vaginal fluid [34, 50, 100] and whose chemical dissociation can be impacted by changes in pH.

The modulation of pH observed to occur along co-cultivation of *Candida* with *L. gasseri* shows that the use of supernatants from bacterial cells may not be a good proxy to study the interaction since the amount of undissociated acids is much higher than the one obtained in a co-culture supernatant creating confounding effects (is the inhibition caused by the lactobacilli or by the accumulation of undissociated organic acids?). The use of bacterial supernatants obtained from cultivation in MRS is particularly problematic since these will invariably contain toxic amounts of acetic acid. In this context, some of the inhibitory effects reported in growth and virulence traits of *Candida* upon exposure to lactobacilli supernatant cultures result, in fact, from the effects of acetic acid. In line with our results, the acetate present in MRS medium was described to have antifungal properties when used in synergy with *Lactobacillus rhamnosus* [101]. Collectively these findings increase the relevance of using experimental settings based on contact of the species as they appear to establish dynamic interactions that may go beyond the mere accumulation of metabolites in the broth and, therefore, are not fully phenocopied by bacterial supernatants.

Co-cultivation of *C. albicans* or *C. glabrata* with *L. gasseri* resulted in decreased viability of the two yeasts (and this is induced by the bacterium and not by accumulated organic acids since we demonstrated that in co-cultures the non-toxic acetate and lactate forms prevailed), either under planktonic biofilm-forming conditions. Previous studies showed that biofilm formation by *C. albicans* is impacted by co-cultivation with the vaginal species *L. crispatus, L. jensenii* and *L. iners* [33, 63], however, as the biofilms formed were not microscopically observed the authors did not concluded about the capacity of these species to interact with one another. Both *C. albicans* and *C. glabrata* had been described to form mixed biofilms with other bacteria including *Streptococcus mutans* [102], *Staphylococcus aureus* [103] or *Pseudomonas aeruginosa* [104], but to the best of our knowledge this is the first report involving *L. gasseri*. In the biofilm, the *C. glabrata* cells were highly susceptible to the presence of *L. gasseri*, while the reduction of viability of *C. albicans* cells was considerably lower (opposite to what was observed under planktonic conditions). We cannot rule out that the mechanism of inhibition prompted by *L. gasseri* in a biofilm may differ from those imparted in planktonic growth as close cell-cell contacts (well demonstrated to occur in **Fig. 2**) may trigger specific responses. In this context, the previous demonstration that *L. paracasei* cells respond to direct contact with *S. cerevisiae* cells is interesting [105]. Another hypothesis is that the access of *L. gasseri* to *C. glabrata* cells in the mixed biofilm can be higher due to a stiffer structure of the *C. albicans* biofilms caused, among other aspects, by high amounts of extracellular matrix [106, 107]. The higher sensitivity of *C. glabrata* to *L. gasseri* in the mixed biofilm is interesting since this species is much less frequently isolated from the vaginal tract than *C. albicans* [7, 108, 109], although it is known to be very high resilient to environmental stress [9, 54, 110].

The fact that the anti-*Candida* activity prompted by *L. gasseri* cells depends on the presence of acetate in a concentration-dependent manner is a novel finding of our work. *In vivo* acetate is present in the vaginal fluid due to metabolic activity of colonizing microbes [50, 111, 112] and thus it is possible that it contributes to maintain the interference of *L. gasseri* over *Candida*. Note that the potential of acetate in augmenting virulence of *L. gasseri* towards *Candida* cells was detectable at 4 mM acetate, a concentration within the range found in vaginal fluid in a lactobacilli-dominated vaginal microflora [50, 113, 114]. Little is known concerning the biology and physiology of *L. gasseri* and thus the effects of acetate remain to be studied. In some Gram positive species such as *L. plantarum, Lactobacillus sakei, Lactobacillus plantarum* and *L. rhamnosus,* acetate potentiated the expression of bacteriocin-encoding genes [91, 115-117] and thus one possibility is that it may have a similar effect in *L. gasseri*. Due to their small size and significant inter-species variation, the annotation of bacteriocin-encoding genes is difficult [118, 119]. Recent genomic analysis of *L. gasseri* strains (including ATCC33323) predicted that this species encodes acidocin A, gassericin and helveticin J [67, 120, 121]. However, in the reference strain only helveticin J, a class III bacteriocin that has been shown to have some activity against two clinical strains of *C. albicans* and *C. glabrata* [67], is represented in its genome. The fact that cellular density also affects the modulatory effect of acetate over bacteriocin-encoding genes [91, 117, 122] creates an important factor that has to be considered in a future study that may aim the study of the molecular mechanism underlying this acetate-induced virulence of *L. gasseri* against *Candida*.

## MATERIALS AND METHODS

### Strains and growth media

In this study we used the reference strains *L. gasseri* ATCC33323 (acquired from DSMZ); *C. glabrata* KUE100 (a wild-type strain derived from the CBS138 strain [123]); and *Candida albicans* SC5314 strains. We have also made use of five clinical strains: *L. gasseri*, ISTLg97, a vaginal isolate whose species identity was confirmed by Maldi-TOF and based on sequencing of the 16S RNA sequence; *C. glabrata* VG49, *C. glabrata* VG216, *C. albicans* VG217 and *C. albicans* VG485, all vaginal strains that had been recovered along epidemiological surveys undertaken in the Lisbon area [124]. The MRS medium used to co-cultivate yeasts and bacteria contains, per liter 10 g casein peptone (Gibco), 10 g meat extract (Panreac AppliChem), 5 g yeast extract (Gibco), 20 g glucose (Nzytech), 1 g Tween 80 (Sigma), 2 g K_2_HPO_4_ (Merck), 5 g sodium acetate (Merck), 3 g ammonium sulphate (Panreac AppliChem), 0.20 g MgSO_4_.7H_2_O (Labchem) and 0.05 g MnSO_4_.H_2_O (Sigma). After preparation, pH of MRS was adjusted to 6.2-6.5 using HCl or NaOH. In indicated experiments the sodium acetate used to prepare MRS was replaced by sodium chloride (Honeywell, Fluka^TM^). YPD medium, used for maintenance of the strains, contains, per liter 20 g glucose (Nzytech), 20 g peptone (Gibco) and 10 g yeast extract (Gibco). Solid YPD or MRS were prepared by supplementing the corresponding liquid medium with 2% and 1.5% agar (Nzytech), respectively. Media were prepared using deionized water and sterilized by autoclaving for 15 min at 121°C and 1 atm.

### Co-cultivation in liquid MRS medium of *L. gasseri* with *C. glabrata* or *C. albicans*

To examine growth of *L. gasseri* in liquid MRS alone or in the presence of *C. glabrata* or *C. albicans*, a pre-inoculum of each individual species was prepared in MRS and the cells were cultivated, overnight, at 37°C with an orbital agitation of 100 rpm. On the next day, these cells were used to inoculate (at an OD_600nm_ of 0.4 for *L. gasseri* and 0.1 for the two *Candida* species) fresh MRS medium. Growth in this co-culture system was accompanied for 4 days at 37°C and using an orbital agitation of 100 rpm, by following the increase in cellular viability of the two species based on the number of colony forming units (CFUs). For this, aliquots of co-cultures were taken, serially diluted and plated on MRS supplemented with 96 mg/L fluconazole (an antifungal concentration that fully prevented growth of *Candida* colonies and thus only *L. gasseri* colonies were visible) or in YPD supplemented with 300 mg/L tetracycline (an antibiotic concentration that fully prevented growth of *L. gasseri* colonies and therefore only *Candida* colonies were visible). The number of *Candida* colonies formed on the surface of YPD plates was counted after 2 days of incubation at 30°C, while the number of *L. gasseri* colonies formed onto the surface of MRS plates was counted after 2 days of plate incubation at 37°C in a Genbox (Biomerieux) with a candle inside to assure microaerophilia [125, 126]. As controls we performed single-cultivations of *L. gasseri, C. albicans* and *C. glabrata* under the same conditions used for the co-cultivations. Quantification of the amounts of lactic acid, acetic acid or glucose present in the broth during single or multi-species cultivation was performed by HPLC (equipped with an UV detector, for quantification of lactic and acetic acids, and with an RI detector, for quantification of glucose) using an Aminex HPX87H (Biorad®) column and 0.005M H_2_SO_4_ (at a flow rate of 0.6 mL/min of) as eluent.

### Co-cultivation of *L. gasseri* with *C. glabrata* or *C. albicans* under biofilm-forming conditions

To examine growth under biofilm-forming conditions of *L. gasseri* alone or in co-cultivation with *C. albicans* or *C. glabrata*, a pre-inoculum of each individual species was prepared in MRS (or in this same medium containing sodium chloride as a sodium source) and the cells were cultivated overnight at 37°C using an orbital agitation of 100 rpm. These pre-cultures were used to inoculate 200 µL of fresh MRS in plastic µ-slide 8 well plates (Ibidi) so that the initial cell densities (estimated based on OD_600nm_) were 10^6^ CFU/mL for the two *Candida* species and 2x10^8^ CFU/mL for *L. gasseri*. After 24 h of cultivation at 37°C with 25 rpm agitation, the supernatant of the single or co-cultures was removed and the biofilm formed washed with 200 µL of PBS. In order to assess cellular viability in the single- or multi-species biofilms formed, 3 μM of SYTO 9 Green Fluorescent Nucleic Acid Stain (Molecular Probes, Eugene, OR, USA) was added to the single or co-cultures and the cells were left in the dark for 30 minutes. After this time, 4 μM TO-PRO-3 iodide (Molecular Probes, Eugene, OR, USA) were added and the cultures were incubated under the same conditions for another 15 minutes. The gain adjustment in each channel was optimized (and kept during the experiments) taking into account the intensity of the fluorescence signal of live and dead single cells. Live single cells were stained directly after growth, while dead single cells were prepared by heating a cell sample at 65°C for 10 minutes in a dry bath. Then, single or multiple species biofilms were imaged by confocal laser scanning microscopy using a Leica TCS SP5 inverted microscope with a 63x water (1.2 numerical aperture) apochromatic objective. Cells were imaged with the 488 nm Ar^+^ laser line to detect cells stained with SYTO 9 (emission collected at 500 – 590 nm) and with the 633 nm He-Ne laser line to detect cells stained with TO-PRO-3-Iodide (emission collected at 645-795 nm), a setup that minimizes cross interference between the two channels as described in [127]. To measure the height of the biofilms, the same experimental setup was used with the difference that the cells were only labelled with SYTO9 and the confocal microscopy images used for height quantification.

## SUPPLEMENTAL MATERIAL

Click here for supplemental data file.

All supplemental data for this article are available online at http://www.microbialcell.com/researcharticles/2023a-pedro-microbial-cell/.

## Abbreviations:

NACS: – non-albicans Candida species.
