# Supplementary material for: Acetate modulates the inhibitory effect of Lactobacillus gasseri against the pathogenic yeasts Candida albicans and Candida glabrata
Source: Microb Cell. 2023 Mar 21;10(4):88–102. doi: 10.15698/mic2023.04.795 (PMC10054710; doi:10.15698/mic2023.04.795)
Supplement: Supplementary file 1 [file mic-10-088-s01.pdf]

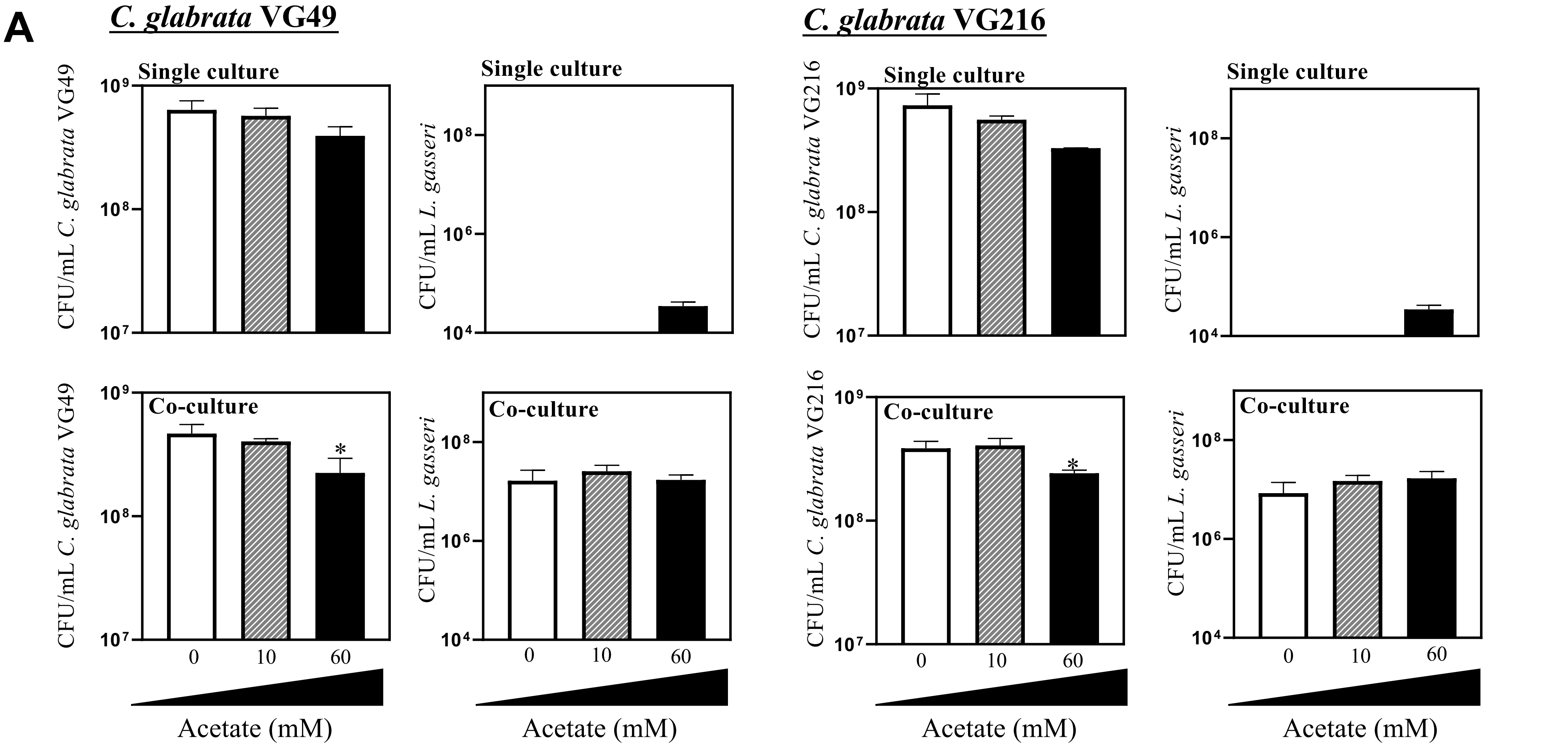

Figure S1 – Viability of vaginal *C. glabrata* (A) and *C. albicans* (B) vaginal strains after 96h of single or co-cultivation with *L. gasseri* in MRS medium (corresponding to a concentration of acetate of 60 mM) or in this same medium having 60 mM NaCl (in replacement of the sodium acetate) or 10 mM sodium acetate and 50 mM NaCl

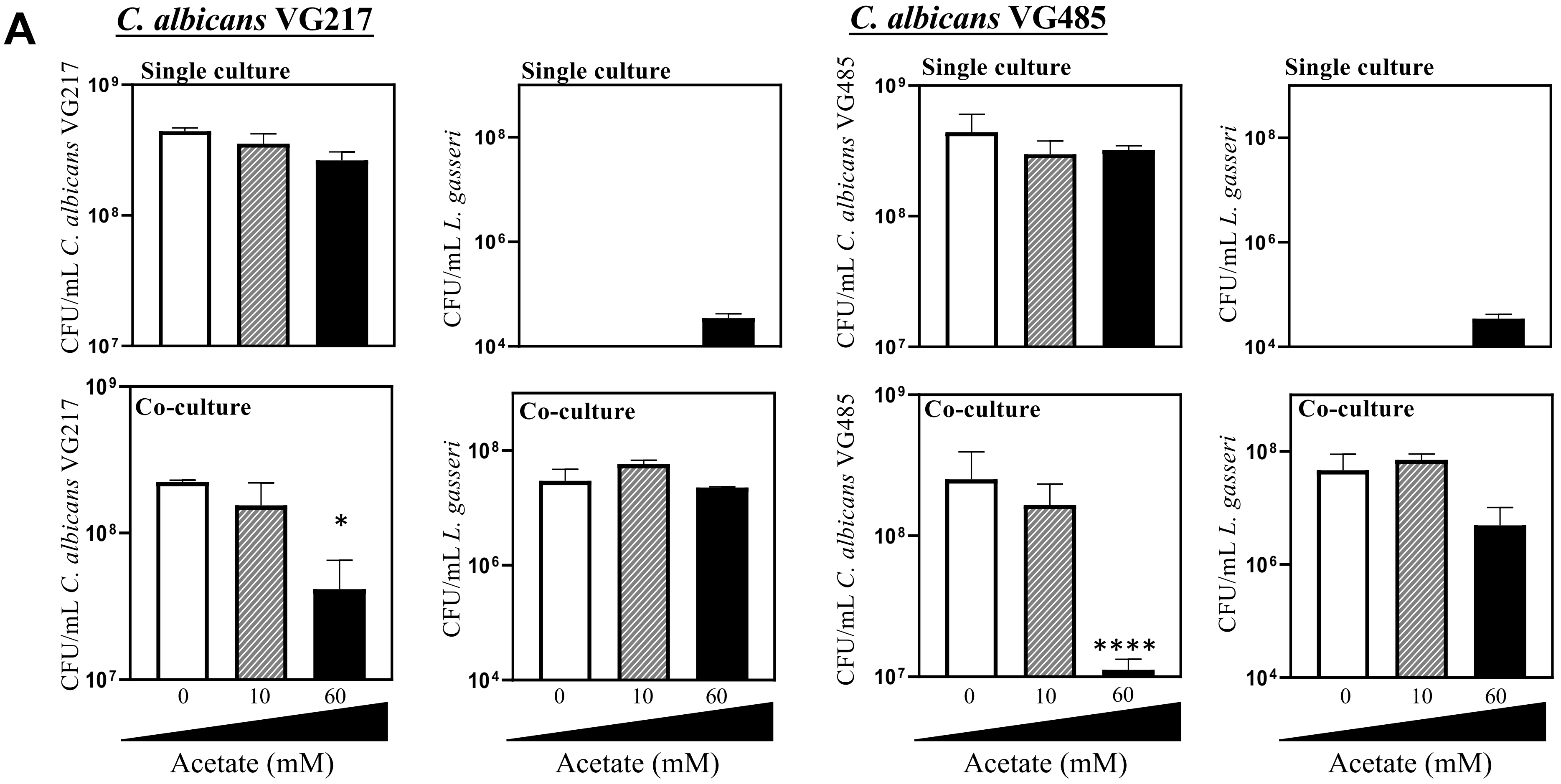

Figure S1 – Viability of vaginal *C. glabrata* (A) and *C. albicans* (B) vaginal strains after 96h of single or co-cultivation with *L. gasseri* in MRS medium (corresponding to a concentration of acetate of 60 mM) or in this same medium having 60 mM NaCl (in replacement of the sodium acetate) or 10 mM sodium acetate and 50 mM NaCl

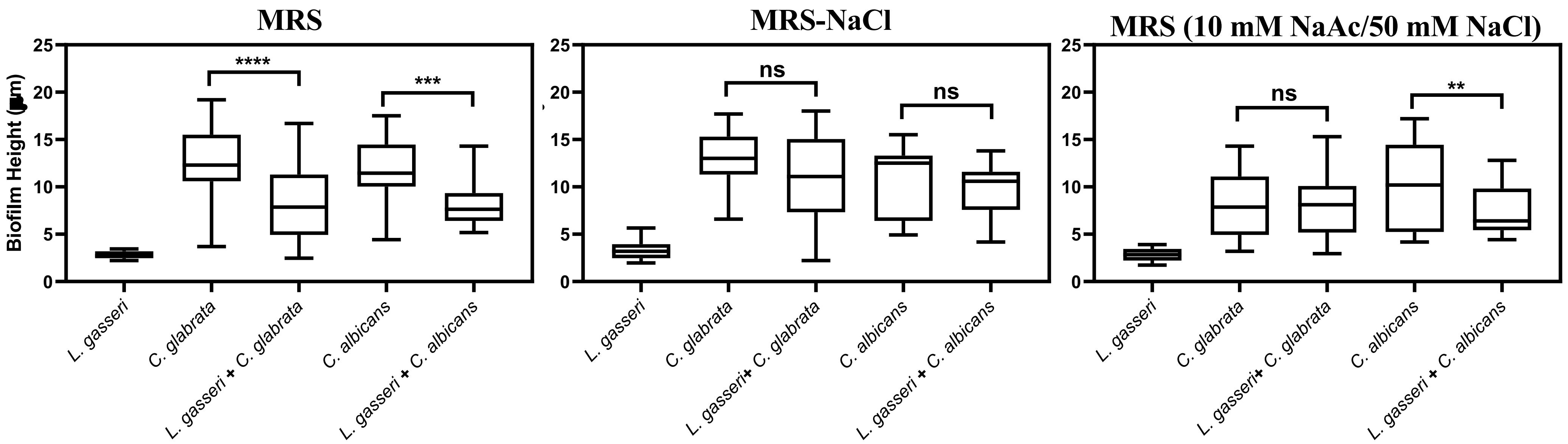

Figure S2 – Height of the biofilms (in µm) formed by *L. gasseri*, *C. albicans* and *C. glabrata* when cultivated, for 24h, alone or in combination in MRS medium containing 60 mM of NaCl (in replacement of sodium acetate) or containing 10 mM sodium acetate (NaAc) and 50 mM NaCl. After cultivation in 8-well microplates, the single and multi-species biofilms were washed, labelled with SYTO9, fixed and imaged by confocal microscopy, as detailed in materials and methods.

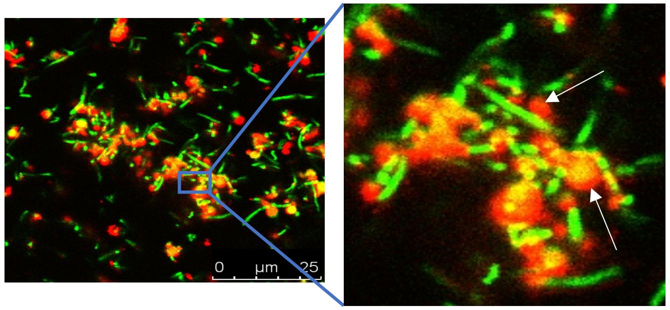

Figure S3 – Detail on the imaging obtained after the overaly of the SYTO9 and To-Pro3-Iodine labelling of biofilms formed by *L. gasseri* after 24h of cultivation in MRS medium, as detailed in Fig.2. Note the marked red accumulation outside of the bacterial cells, in what appears to be the extracellular matrix of the biofilm. These images were obtained after 24h of cultivation at 37ºC and 25 rpm. Scale bar corresponds to 25 μm;

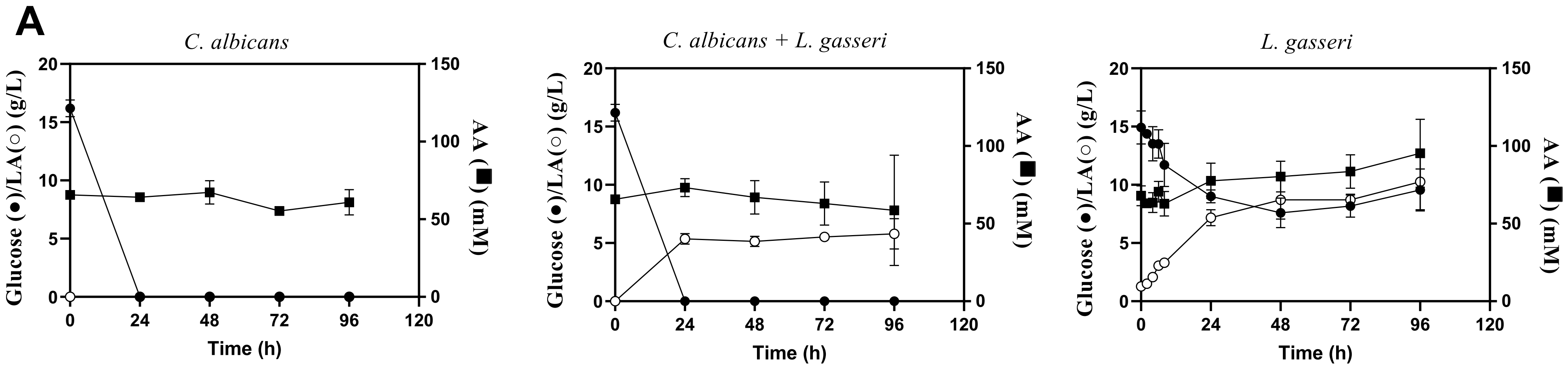

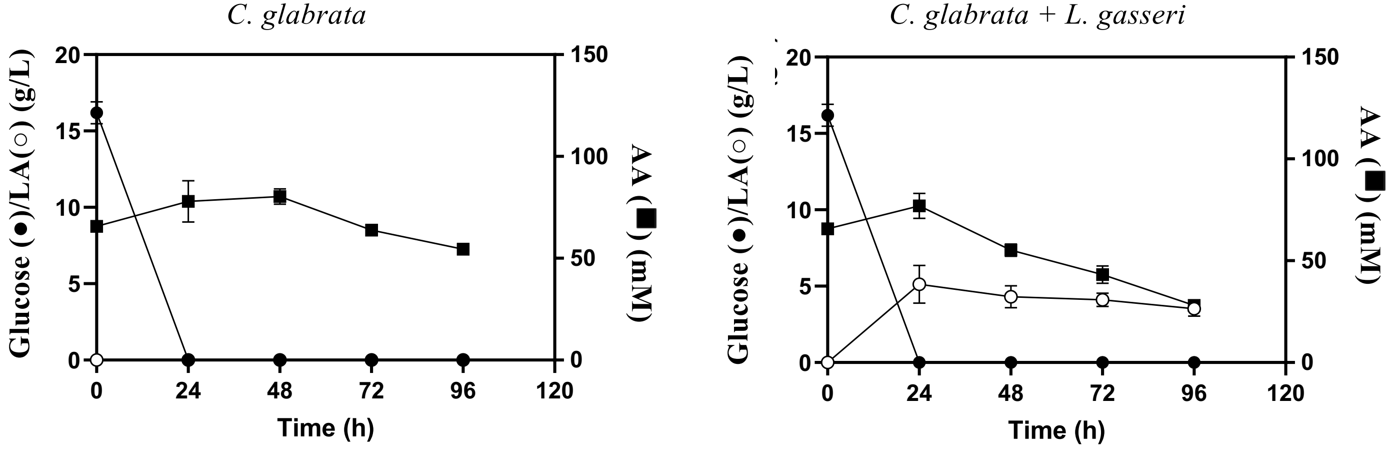

Figure S4 (A). Quantification of glucose (●), lactic acid (LA) (○) and acetic acid (AA) (■) in supernatants of cultures obtained upon cultivation of *L. gasseri,* alone or in combination with *C. glabrata* or *C. albicans,* in MRS medium. The results obtained correspond to those obtained in three independent replicas.

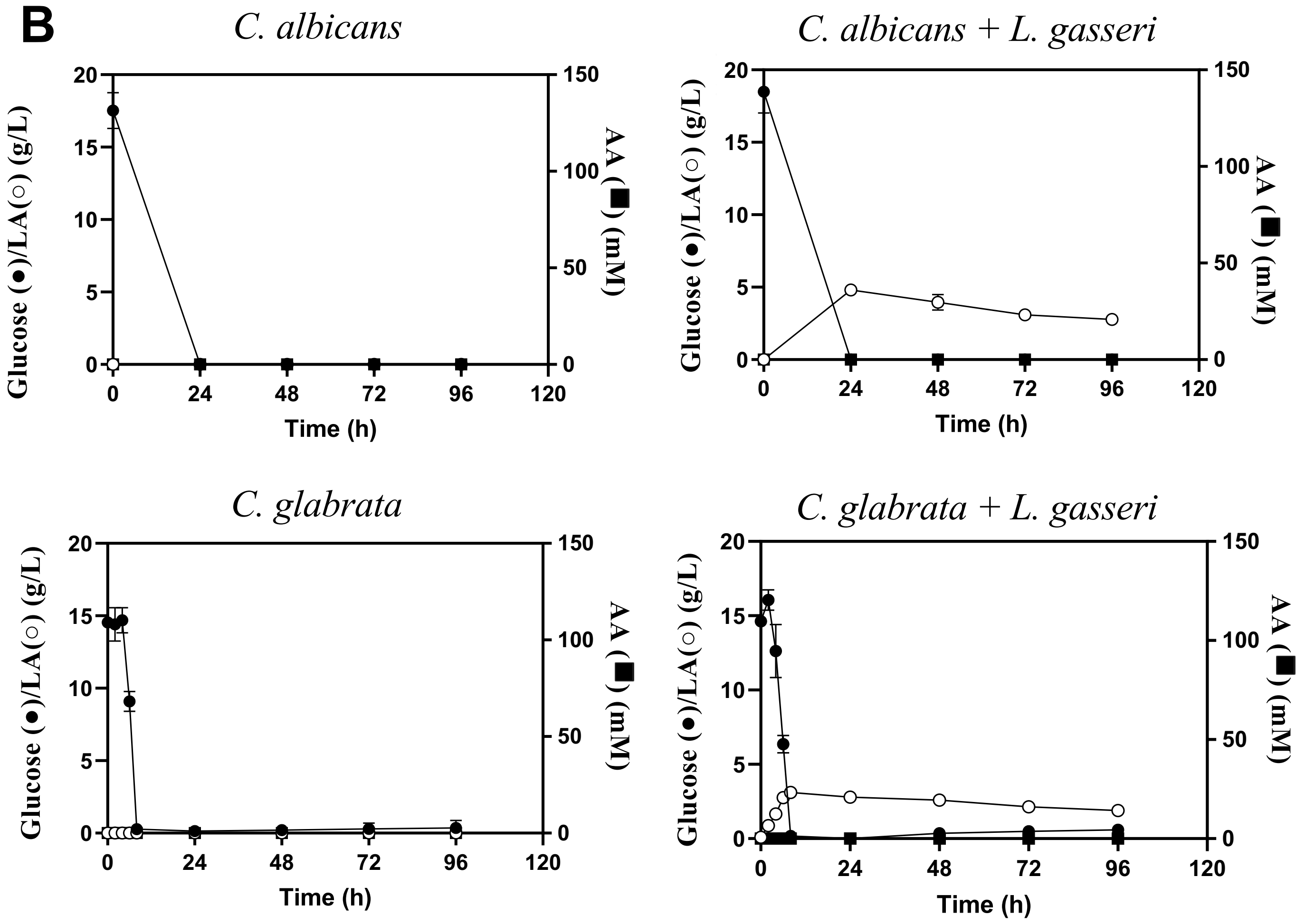

Figure S4 (B) Quantification of glucose (●), lactic acid (LA) (○) and acetic acid (AA) (■) in supernatants of cultures obtained upon cultivation of *L. gasseri,* alone or in combination with *C. glabrata* or *C. albicans,* in MRS-NaCl medium. The results obtained correspond to those obtained in three independent replicas.

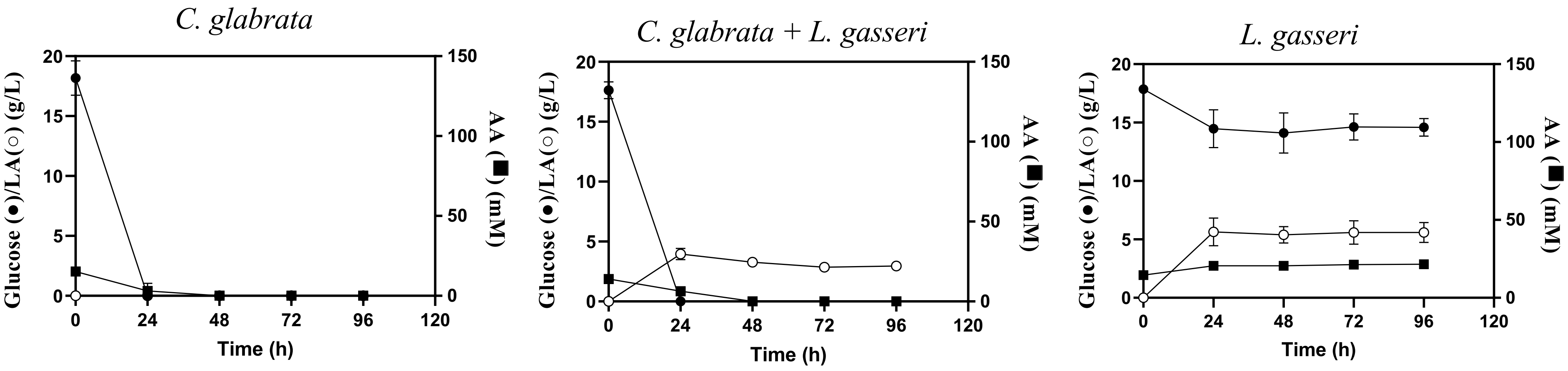

Figure S4 (C) Quantification of glucose (●), lactic acid (LA) (○) and acetic acid (AA) (■) in supernatants of cultures obtained upon cultivation of *L. gasseri,* alone or in combination with *C. glabrata* or *C. albicans,* in MRS having 50 mM NaCl and 10 mM sodium acetate. The results obtained correspond to those obtained in three independent replicas.

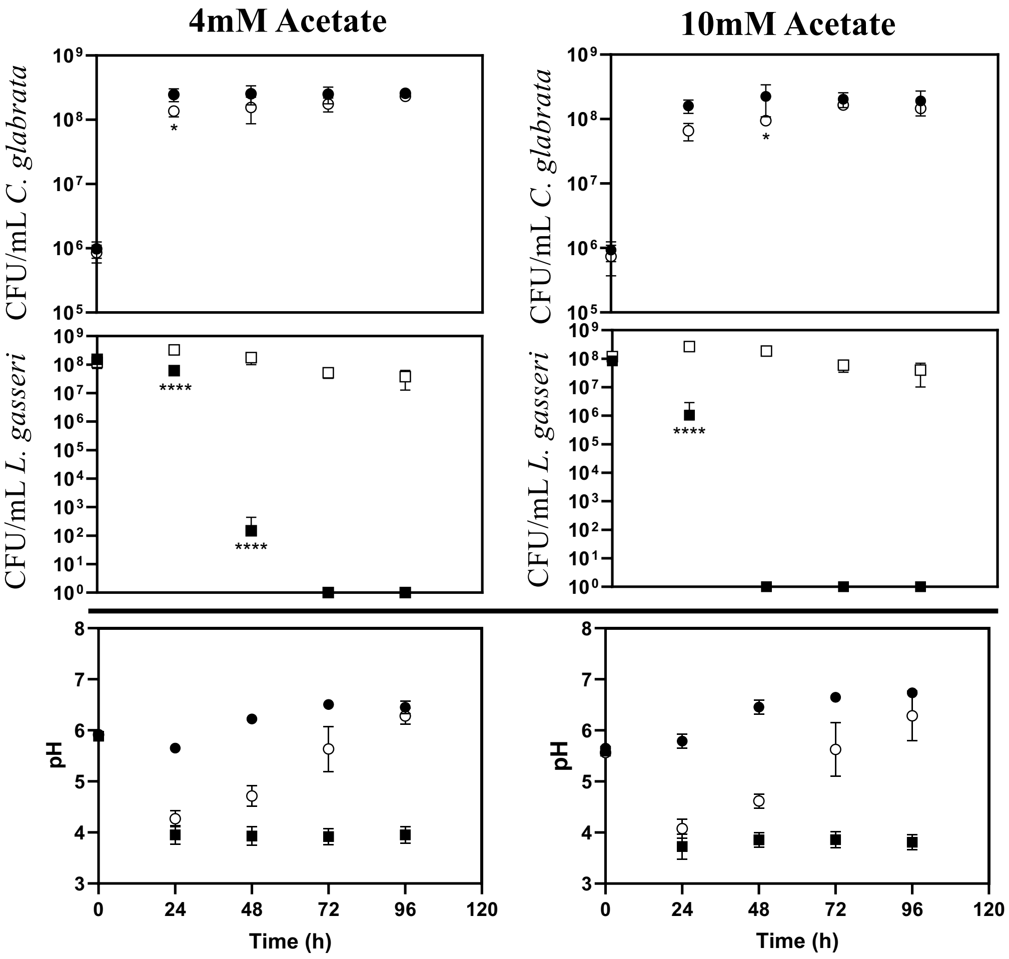

Fig. S5. Cellular viability and medium pH during single or co-cultivation of *C. glabrata* (○,●) with *L. gasseri* (□,■) in MRS medium having 4 or 10 mM acetate (supplied in the form of sodium acetate). To maintain the total of amount of sodium in 60 mM (the amount present in canonical MRS medium), we have further supplemented the MRS medium with 56 or 50 mM sodium chloride. The cells were cultivated, alone or in the presence of each other, in the same conditions described in Fig.1. Filled symbols correspond to the samples taken during single-species cultivation while open symbols corresponds to the samples taken during co-cultivation.;

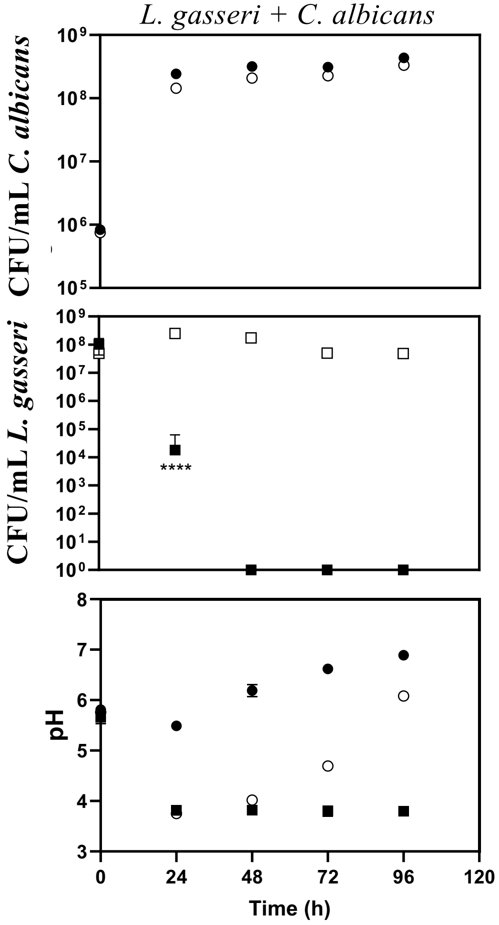

Fig. S6. Cellular viability and medium pH during single or co-cultivation of *C. albicans* (○,●) with *L. gasseri* (□,■) in MRS medium having 60 mM sodium chloride (instead of the 60 mM sodium acetate present in canonical MRS medium). The cells were cultivated, alone or in the presence of each other, in the same conditions described in Fig.1. Filled symbols correspond to the samples taken during single-species cultivation while open symbols corresponds to the samples taken during co-cultivation.; Statistical significance of the differences found in the presence or absence of acetate were calculated using one-way ANOVA (*p-value below 0.05; **p-value below 0.01; ***p-value below 0.001; ****p-value below 0.0001).

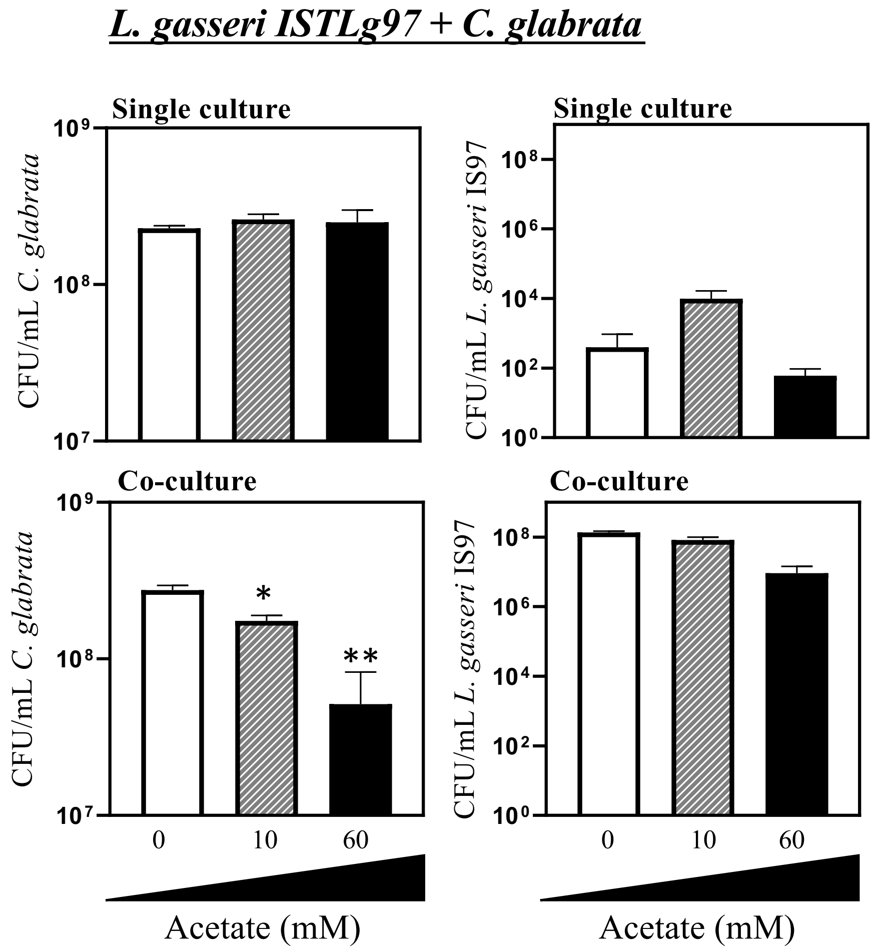

Fig. S7 - Viability of *C. glabrata* after 96h of single or co-cultivation with the vaginal clinical *L. gasseri LgIST97* in MRS medium (corresponding to a concentration of acetate of 60 mM) or in this same medium having 60 mM NaCl (in replacement of the sodium acetate) or 10 mM sodium acetate and 50 mM NaCl
